# Optimization of Dry-Jet Wet Spinning of Regenerated Cellulose Fibers Using [mTBDH][OAc] as a Solvent

**DOI:** 10.1021/acsomega.3c05133

**Published:** 2023-08-29

**Authors:** Wenwen Fang, E Yee Lim, Kaarlo Leo Nieminen, Herbert Sixta

**Affiliations:** Department of Bioproducts and Biosystems, Aalto University, Vuorimiehentie 1, 02150 Espoo, Finland

## Abstract

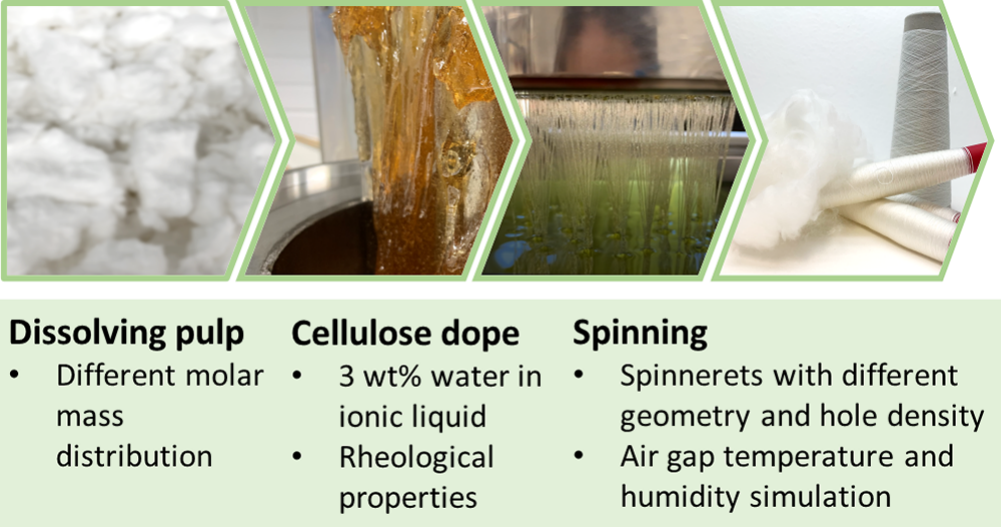

Superbase-based ionic liquids (ILs) have demonstrated excellent dissolution capability for cellulose, and employing the dry-jet wet spinning process, high-tenacity regenerated textile fibers have been made. Among a range of superbase-based ILs, [mTBDH][OAc] exhibited not only good spinnability but also exceptional recyclability, making it highly suitable for a closed-loop production of regenerated cellulose fibers. To further optimize the spinning process, we investigated the influence of the cellulosic raw materials and the IL with residual water on spinnability and fiber properties. In addition, single-filament spinning and multifilament spinning using spinnerets with different hole densities were investigated to reveal the upscaling challenges of the dry-jet wet spinning process. The air gap conditions, for example, temperature and moisture concentration were simulated using COMSOL multiphysics. The results indicate that the presence of a small amount of water (3 wt%) in the IL has a positive effect on spinnability, while the mechanical properties of the fibers remain unchanged.

## Introduction

In recent years, the impact of climate change and extreme weather events has intensified discussions around transitioning to a sustainable economy. Despite this, the demand for textiles continues to increase due to population growth and the popularity of fast fashion. According to the Global Textile Fibers Market Report 2021–2027, global textile consumption reached 109.5 million tons in 2020, and it is estimated to keep growing at an annual rate of 3%. However, until 2018, only 35.8% of textiles were produced from natural fibers, including 24.1% cotton, 6.2% wood-based cellulosic fiber, and other natural fibers.^1^

Cotton, often thought of as an environmentally friendly material, is actually grown in inherently dry areas, leading to the exploitation of scarce water sources. The Circle Economy estimates that the cultivation of cotton consumes 25% of the world’s insecticides, 10% of pesticides, and as much as 2.5% of global water.^2^ A sustainable alternative is offered by man-made cellulosic fibers (MMCFs) produced from abundant cellulose resources. The textile industry primarily relies on viscose and lyocell fibers, which suffer from environmentally harmful processes and solvents or potential safety concerns from runaway reactions, highlighting the need for greener and safer alternatives.

In 2002, Rogers and coworkers rediscovered ionic liquids (ILs) and demonstrated their powerful dissolution capabilities for cellulose.^3^ Since then, there has been a renaissance in designing specific ILs for cellulose processing.^4,5^ The unique dissolution capacity of ILs is due to their ability to disrupt the inter- and intramolecular hydrogen bonds and van der Waals interactions between cellulose chains. The anions of the ILs form strong hydrogen bonds with the hydroxyl groups of cellulose, while the cations are in close proximity to the anion–cellulose complex, providing steric repulsion within the complex.^6^ It is believed that hydrophobic interactions between the cations and cellulose also play a crucial role in cellulose dissolution.^7^

Most of the reported cellulose-dissolving ILs were imidazolium-based and all included an anion with a high hydrogen-bond basicity, such as chlorides and carboxylates.^5,8^ Imidazolium-based ILs have generated significant interest because they can dissolve cellulose at high concentrations, and imidazolium acetate in particular has low viscosity, which facilitates the handling and dissolution of cellulose.^9^ However, it has been demonstrated that cellulose undergoes significant degradation depending on the substitution of the imidazolium ring and anions used, especially at high temperatures (>90 °C).^10−12^ Additionally, the degradation of imidazolium-based ILs, such as [EMIM]Cl and [BMIM]Cl, at relatively low temperatures, around 120 °C, makes the recycling of the IL challenging and thus limits its application on a large scale.^13^

To address this issue, superbase-based ILs have been developed, which exhibit excellent cellulose dissolution capacity at relatively low temperatures, thus preventing cellulose degradation.^14,15^ In addition to efficient dissolution, cellulose dissolved in superbase-based ILs shows suitable viscoelastic properties for dry-jet wet spinning. As a result, a new process called Ioncell-F technology has been developed for producing regenerated cellulose fibers.^14^ Because of the milder process conditions, the cellulose is less degraded, resulting in higher fiber yield and improved mechanical strength. It enables the direct incorporation of functional additives into the fiber structure by dissolving and extruding them together with cellulose. For instance, UV-sensitive, antibacterial, and hydrophobic properties have been imparted to the fibers.^16−18^ Importantly, this technology allows for the production of cellulose fibers with excellent mechanical properties using various cellulosic waste sources, including paperboard and recycled cotton.^19,20^

Among the superbase-based ILs used in the Ioncell process, 7-methyl-1,5,7- triazabicyclo[4.4.0] dec-5-enium acetate ([mTBDH][OAc]) has been identified as the most promising IL for commercial use due to its excellent thermal and hydrolytic stability.^21,22^ It can be easily separated and recycled from aqueous solutions through distillation, with only 2–3 wt % of hydrolysis compounds and approximately 3 wt % of water accumulating in the residual IL after five recycling cycles.^22^ Furthermore, [mTBDH][OAc] exhibits high tolerance to impurities such as water and hydrolysis products and can achieve a dissolution rate higher than 99% even in the presence of 1 wt % water and 20 wt % hydrolyzed mTBD, making it a promising solvent for industrial use.^21^ Although we have demonstrated successful spinning using [mTBDH][OAc] as a solvent, the spinning parameters have not been fully explored. Spinning trials have mainly been performed using single filament spinning or multifilament spinning with a low capillary density spinneret. However, industrial spinning lines typically use spinnerets with high capillary density, and air gap conditioning significantly differs from that of single filament spinning. Therefore, it is crucial to stabilize the spinning process and understand the key parameters that influence spinnability in order to prepare for the commercialization of the ioncell process.

The molar mass distribution (MMD) of cellulose plays a crucial role in determining the viscoelastic properties of the cellulose dope, which in turn affects the spinnability and fiber properties.^23,24^ Acid sulfite (AS) pulp and prehydrolysis kraft (PHK) pulp are the commonly used dissolving pulps for regenerated cellulose fiber spinning, both containing more than 90 wt % of α-cellulose. Previously, PHK pulp was primarily used as the cellulose resource, which showed excellent performance when 1,5-diaza-bicyclo[4.3.0]non-5-enium acetate ([DBNH][OAc]) was used as the solvent. However, the spinnability was not very stable when [mTBDH][OAc] was used as the solvent. It is assumed that AS pulp, which has a broader MMD and a higher content of high *M*_w_ fraction, could enhance the spinnability. Another observation in our previous spinning trials is that the recycled [mTBDH][OAc] containing a small amount of residual water and hydrolysis products showed improved spinnability compared with fresh prepared IL (not published data). This unexpected finding suggests that the presence of residual water may alter the viscoelastic properties of cellulose dope, which can in turn affect the spinnability.

In this study, we will be using AS dissolving pulps with different MMD to conduct spinning trials using [mTBDH][OAc] as the solvent. To investigate the effect of residual water from IL recycling on dope spinnability, a small amount of water will be added to freshly prepared IL. Additionally, we will explore the challenges associated with upscaling the spinning process by examining spinnability and fiber properties using spinnerets with different geometries and hole densities.

## Materials and Methods

### Pulp

In this work, two different spruce AS dissolving pulps with varying intrinsic viscosities were received in the form of sheets from AustroCel Hallein (Austria). The sheets were ground using a Wiley mill, and the dry matter content was determined before dissolution in an IL.

### Ionic Liquid

7-Methyl-1,5,7-triazabicyclo[4.4.0]dec-5-enium (mTBD, Haoyuan Chemexpress Co., China) was neutralized with an equimolar amount of acetic acid (OAc, glacial, 100%, Merck, Germany) to form [mTBDH][OAc]. The neutralization process was carried out at 80 °C to prevent crystallization of the IL. The mixture was stirred for an additional 30 min to ensure complete conversion to [mTBDH][OAc]. A 3 wt% water IL was prepared by adding 3 wt% water to the freshly prepared IL before the dope preparation.

### Dope Preparation

The cellulose dope was prepared using a vertical kneader at 85 °C with stirring (30 rpm) and vacuum (30–40 mbar) for approximately 120 min. The resulting cellulose solution was then filtered using a heated hydraulic press filtration system (85 °C, 150–200 bar, 5–6 μm mesh metal filter fleece). The dope was then molded, tightly wrapped with parafilm to prevent moisture exchange, and stored at 4 °C.

### Rheology Measurement

The rheological properties of cellulose dopes were measured using an Anton Paar MCR 302 rheometer equipped with a 25-mm diameter parallel plate geometry. The measuring gap was kept at 1 mm. Dynamic frequency sweeps were performed from 0.01 to 100 s^–1^ at elevated temperatures ranging from 60 to 100 °C in the linear viscoelastic region (LVR) with a constant amplitude strain of 0.5%. The zero-shear viscosity, η°, was determined by fitting the complex viscosity data to the cross viscosity model.

### Monofilament Spinning

A small dope (20–35 g) was inserted into a monofilament dry-jet wet spinning unit (Fourné Maschinenbau GmbH) and heated up to its spinning temperature estimated according to the rheological measurements. The diameter of the capillary is 100 μm, and the length is 200 μm. The dope was extruded through an air gap (1 cm) into a water coagulation bath (approximately 6 °C) where the cellulose was regenerated. The detailed description of the single filament spinning line could be found in our previous publication.^25^ The extrusion velocity was kept constant at 1.3 m/min, while the take-up velocity (the speed of the godets collecting the fibers) was varied to collect fibers at different draw ratios (DRs). The collected filaments were cut and washed with 80 °C water for 2 h to remove the residual IL.

### Multifilament Spinning

The spinning process for the multifilament spinning was similar to that of the monofilament spinning, but spinnerets with different capillary number, density, and geometry were used. One spinneret with circular geometry (400 holes × 100 μm diameter × 20 μm length) and two spinnerets with rectangular geometry (400 × 100 × 20 and 504 × 100 × 100) were used. The distribution of the holes in the spinnerets is illustrated in Figure S1 (Supporting Information). The amount of dope used for spinning the multifilament fibers was much larger, ranging from 1.2 to 1.6 kg.

### Modeling of Temperature and Moisture Distributions in the Air Gap

The stationary temperature and moisture distributions in the air gap were simulated using the COMSOL Multiphysics software employing the modules transport in diluted species as well as heat transfer in fluids. Following the finite element method, the software divides the simulated air gap region into smaller subregions (elements) and numerically solves the differential equations describing the applicable physics. For the modeling of the air gap, the same partial differential equation describes both the diffusion of the moisture (diffusion equation) and the heat transfer (heat equation). In addition, the model must include the transport of water and heat due to the downward movement of the filaments during spinning. Finally, the necessary boundary conditions of the differential equations are the temperature and air humidity at a distance from the filament as well as the temperature and humidity for the filaments exiting the spinneret capillary.

In the case of a single filament, it is possible to take advantage of the circular symmetry of the geometry and reduce the original 3D problem to a 2D problem by calculating the temperature and water content as functions of the radial distance from the central axis of the filament, thereby significantly decreasing the computational effort. As for the simulations of bundles of filaments, it is not possible to deploy full circular symmetry. Instead, the simulation was conducted in two steps, first a simulation of the entire bundle, where the element mesh density at filament level is clearly lower than in the single filament simulation. After observing that the simulated temperature and moisture distributions had horizontal gradients close to zero, the single filament simulation was repeated, this time with the boundary values obtained from the simulation over the bundle of filaments. The filament temperature obtained, which is higher than that for the single filament and the filament water content lower than that for the single filament reflect the altered levels of the distributions in the spaces between the filaments in the bundle. The application of air gap conditioning with an air stream passing through the filament bundle causes the temperature and water contents in the filaments of the bundle to approach the values of the single filament.

### Tensile Testing

The mechanical properties of the spun fibers were evaluated using a Favigraph automatic single-fiber tester (Textechno H. Stein GmbH & Co, Germany) according to ISO 5079 standard. The gauge length was set to 20 mm, the pretension was 0.06 cN/tex, and the test speed was 20 mm/min, with a fiber count of 20. The fibers were conditioned overnight at 20 ± 2 °C and 65 ± 2% relative humidity before the testing. The wet mechanical properties of the fibers were determined by immersing them in water during the tensile test.

### Fiber Birefringence and Total Orientation

The birefringence was measured using a polarized light microscope (Zeiss Axio Scope) equipped with a Berek tilting compensator. For each sample, three filaments were selected for the measurement, and three different spots were measured for each filament.

Birefringence (Δ*n*) was calculated by dividing the optical retardation by the diameter of the fiber, presuming a density of cellulose of 1.5 g/cm^3^. Fiber total orientation (*f*_t_) can then be calculated by dividing Δ*n* by 0.062 (maximum birefringence of cellulose). When *f*_t_ = 0, it indicates the orientation of the fiber is completely random, and likewise, when *f*_t_ = 1, the fiber is perfectly aligned.

### Scanning Electron Microscopy

The morphology of spun fibers was imaged with scanning electron microscopy (SEM, Sigma VP Zeiss). All samples were sputter coated with gold/palladium (80 Au/20 Pd) for 90 s using a Q 150R S plus (Quorum) sputter to improve the conductivity.

## Results and Discussions

### Dissolution of Cellulose in [mTBDH] [OAc]

The spinnability and mechanical properties of regenerated cellulose fibers are significantly influenced by the cellulose raw materials used.^24^ Therefore, this study investigated two AS dissolving pulps with different intrinsic viscosities and MMDs for dry-jet wet spinning using a recyclable IL [mTBDH][OAc]. The chemical composition and macromolecular properties are presented in Table 1. The low-viscosity (LV) Austrocel, which is a standard pulp used for Lyocell process, has an intrinsic viscosity of 489 mL/g. The high-viscosity (HV) Austrocel contains a larger fraction of high-molecular-weight cellulose (21% of DP >2000 cellulose), resulting in a higher intrinsic viscosity of 571 mL/g.

**Table 1:** Chemical Composition, Intrinsic Viscosity, and MMD of HV and LV Austrocel Pulps

	HV austrocel	LV austrocel
chemical composition (%)		
cellulose	95.1	96.1
hemicellulose	4.6	3.6
lignin	0.4	0.4
ash	0.07	0.21
macromolecular properties		
intrinsic viscosity (mL/g)	571	489
*M*_n_ (Da)	56,000	47,000
*M*_w_ (Da)	198,000	164,000
*M*_z_ (Da)	414,000	346,000
PDI	3.5	3.5
DP > 2000 (%)	20.0	14.4
DP < 100 (%)	5.5	6.7

**Figure 1:**
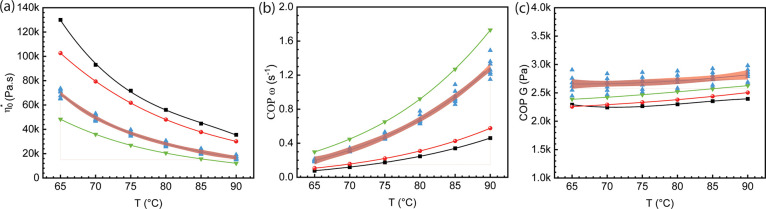
Rheological characterization of dopes prepared from [mTBDH][OAc] with 0.1 wt % water using HV austrocel (black square) and LV austrocel (blue up triangle), and from [mTBDH][OAc] with 3 wt % water using HV austrocel (red sphere) and LV austrocel (green down triangle). (a) Zero shear viscosity (η_0_*), (b) angular frequency (ω) at COP, (c) modulus at COP as a function of temperature. All dopes consist of 13 wt % cellulose. The figure presents polynomial fits and a 95% confidence band, which were applied using Origin software.

The viscoelastic properties of the cellulose dissolution in [mTBDH][OAc], especially the cross-over point (COP), where the storage modulus (*G*’) intersects with the loss modulus (*G*″) and zero shear viscosity (η_0_*), are important parameters to evaluate spinnability.^24^ These properties provide valuable information about the MMD of cellulose and the transition point from more viscous to more elastic cellulose dope.^26^ At low frequencies, the viscous property of the cellulose dope dominates as the molecule has more mobility due to the slow deformation applied. At high frequencies, the entanglement points act as fixed joints and inhibit the flowability of molecules, thus the elastic property dominates. A polymer solution with long molecular chains usually exhibits a low angular frequency at their COP due to the high entanglement density. As it is presented in Figure 1b, the HV Austrocel that has a larger fraction of long molecular chains exhibited a lower ω at COP.

The dissolution of cellulose in IL is achieved by breaking down the strong inter- and intra-molecular hydrogen bond network in cellulose. The dissolved cellulose in IL can be regenerated by the addition of water as a coagulation solvent. The water molecules will first form hydrogen bonds with the free anions in the system and then detach the anions from the cellulose surface and hydrate the hydroxyl groups of cellulose, causing the hydrogen bonds between cellulose chains to reform.

In practical cellulose IL dissolution, a small amount of water is present due to various reasons, such as the initial water content in the pulp, the hygroscopicity of IL, or residue water in recycled IL. As water is also the coagulation solvent in the cellulose regeneration process, it is essential to understand the role of water during cellulose dissolution and its effects on the viscoelastic properties of cellulose dopes. The tolerance of water in cellulose dissolution depends on the IL’s chemical structure. Studies have shown that [mTBDH][OAc] has high water tolerance and can achieve up to 13 wt % cellulose dissolution in the presence of 10 wt % water, while [DBNH][OAc] can only tolerate up to 5 wt % water content.^21^ The addition of a small amount of water has been shown to have a negligible impact on the local environment of cellulose in IL and may even assist the dissolution process as a physicochemical driving force.^6^

In this study, 3 wt % water was added to [mTBDH][OAc] to simulate the residual water remaining in the recycled IL. It is important to note that the pulp utilized in this research has a dry mass content of 96 wt %, implying that in all samples, 0.52 wt % of water originates from the pulp. This applies to both the dopes prepared from freshly synthesized IL, which contains 0.1 wt % water, and IL with an additional 3 wt % water. Despite the dopes being prepared under vacuum, water evaporation from the solution is unlikely to occur based on the vapor–liquid equilibrium study conducted on [mTBDH][OAc] and water.^27,28^ Thermo-recycling of the aqueous solution of [mTBDH][OAc] revealed that the recovered [mTBDH][OAc] contained approximately 2.5–3.5 wt % residual water and 0.5–1 wt % hydrolyzed mTBD in my recent study, which improved the spinning stability. Therefore, an additional 3 wt % water was added to the freshly prepared [mTBDH][OAc] to investigate its impact on the rheological properties of the dope and the resulting fiber properties.

In both HV and LV austrocel dopes, the addition of water resulted in a slight decrease in complex viscosity, while the angular frequency at the COP increased. This phenomenon is consistent with the findings of Hauru et al. who reported that the inclusion of 0.5 equiv water reduces the resilience strength of cellulose solutions in IL.^29^ A recent study by Koide et al. investigated the impact of water on the conformational changes of cellulose dissolved in an imidazolium-based IL ([EMIm][OAc]) using small-angle X-ray scattering. The study suggested that cellulose is surrounded by a layer of interacting solvent molecules, and the addition of a small amount of water (<10 wt %) leads to the formation of complexes with the IL. Interestingly, it was observed that the thickness of this solvent shell decreases with the addition of water, which could explain the decrease in viscosity and elastic properties as the cellulose chain gains increased mobility.^30^

### Monofilament and Multifilament Fiber Spinning

To investigate the impact of pulp composition and residual water in the IL on spinnability and fiber properties, cellulose dopes were prepared from HV and LV austrocel pulps using [mTBDH][OAc] with 0.1 and 3 wt % water. Initially, monofilament spinning was performed, followed by multifilament spinning using spinnerets of varying geometries and hole densities to address upscaling challenges.

**Figure 2:**
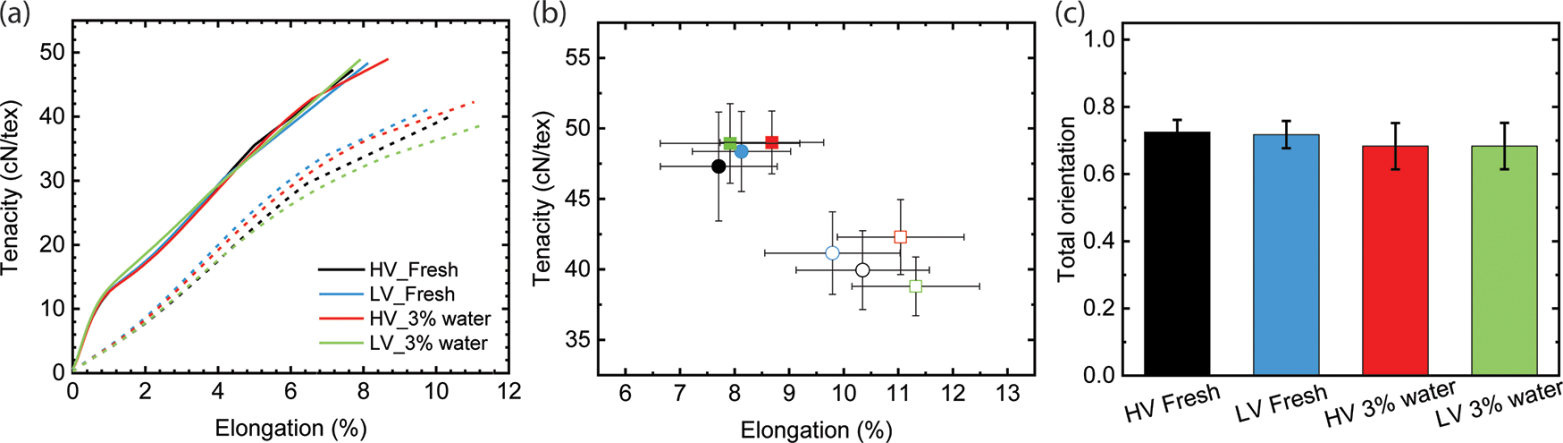
Mechanical properties of fibers spun as monofilaments at DR11. (a) Representative average strain–stress curves of fibers spun from HV and LV Austrocel pulp using [mTBDH][OAc] with 0.1 and 3 wt % water. The fibers were measured under 65% RH (solid lines and symbols) and wet conditions (dash lines and empty symbols). (b) Tenacity as a function of elongation. (c) Fiber orientation calculated from birefringence measurements.

**Figure 3:**
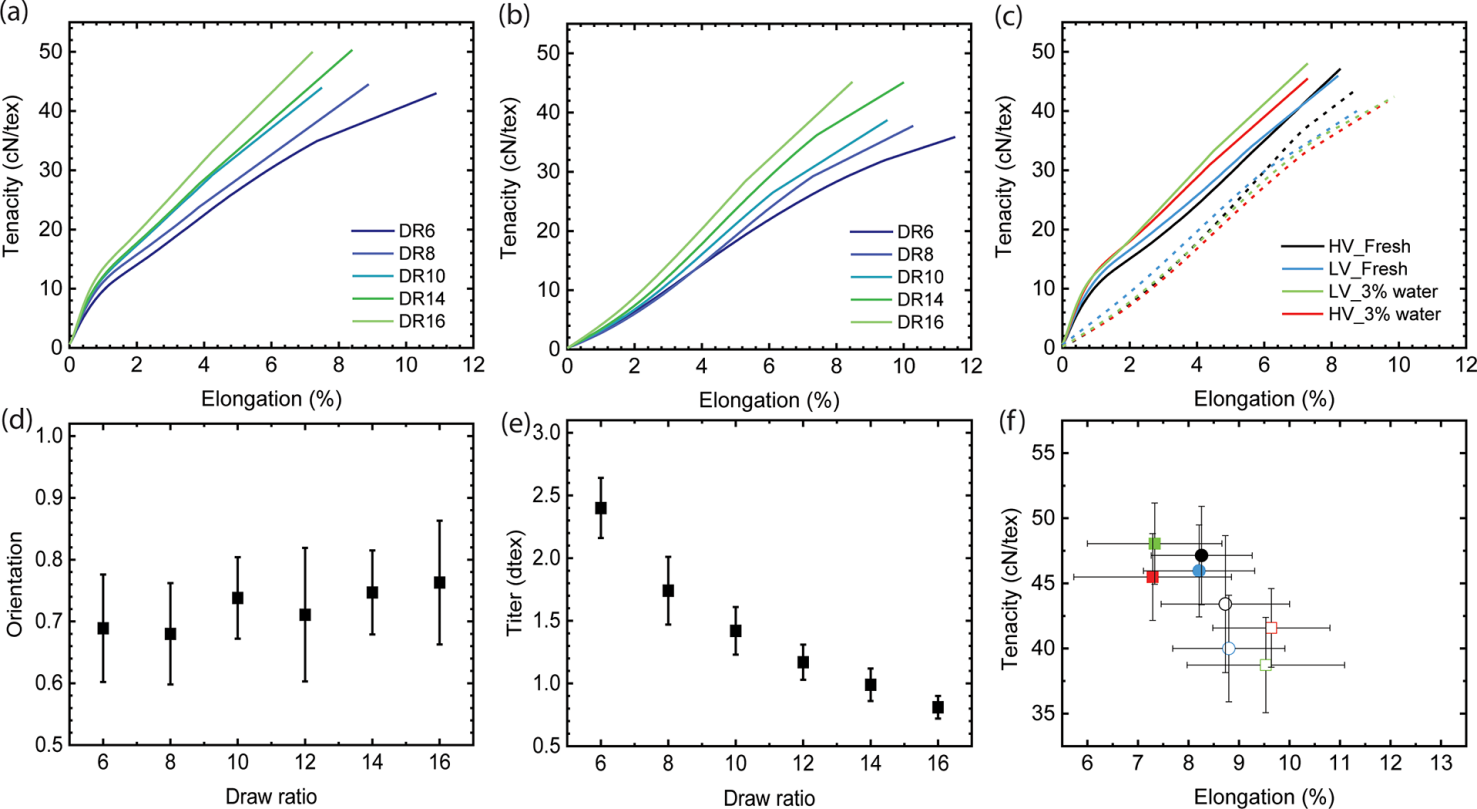
Fiber properties of filaments spun using the multifilament spinning unit and a spinneret with 400 holes. Stress–strain curves of fibers span from HV Austrocel_3 wt % water at different DRs measured at (a) 65% relative humidity (RH) and (b) wet conditions. (c) Stress–strain curves of fibers spun from HV and LV Austrocel pulp using [mTBDH][OAc] with 0.1 wt % water and 3 wt % water at DR 11. (d) Fiber orientation calculated from birefringence measurements. (e) Titer of the fibers. (f) Tenacity plotted as a function of elongation. The fibers are measured at 65% RH (solid lines) and under wet conditions (dashed lines and empty symbols).

Figure 2 illustrates that the mechanical properties of fibers spun from HV and LV Austrocel dissolving pulps are similar despite differences in their viscoelastic properties. However, the presence of a small amount of water (3 wt %) in the IL leads to a slightly higher wet elongation. This can be attributed to lower fiber orientation, as demonstrated in Figure 2c, as the mechanical properties of the filament are highly dependent on fiber orientation. Furthermore, Figure 3 demonstrates that fibers spun at different DRs exhibit significantly varied fiber orientations and mechanical properties. With an increase in DR, the cellulose chains become more aligned within the spinneret capillaries and the air gap, resulting in improved fiber orientation and, subsequently, enhanced mechanical strength (Figure 3). Lower fiber orientation indicates that the entangled cellulose molecules are less aligned within the filament, particularly under wet conditions where water can act as a lubricant for the cellulose chains.

Monofilament spinning serves as a valuable tool for investigating the impact of dope properties on spinning, independent of process conditions such as heat distribution in the spinning dope or air gap conditions. The cellulose dope exhibits poor thermal conductivity due to the relatively low thermal conductivities of cellulose and [mTBDH][OAc], which are approximately 0.1 and 0.16 W m^–1^ K^–1^ at 70 °C, respectively.^31,32^ Therefore, achieving uniform heat distribution within the dope poses a challenge during scale-up.

**Figure 4:**
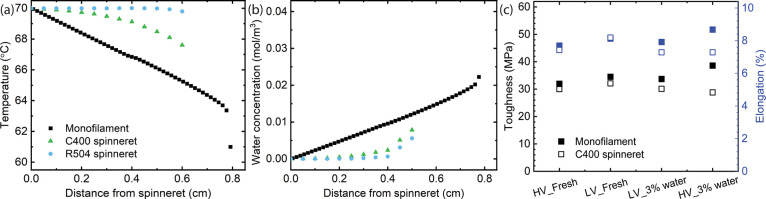
Simulation of temperature (a) and humidity (b) gradient from the spinneret to the coagulation water bath surface at the air gap. This simulation is conducted using different spinnerets, namely the 400-hole circular spinneret (C400) and the 504-hole rectangular spinneret (R504). The air gap distance is set at 0.8 cm. (c) A comparison of the toughness and elongation between monofilament spinning and multifilament spinning. The toughness is calculated by integrating the area under the tenacity-elongation curve. The fibers were collected at DR11. HV and LV represent the high viscosity and low viscosity austrocel pulp with the intrinsic viscosity of 571 and 489 mL/g, respectively.

Another significant change when transitioning from monofilament to multifilament spinning is the air gap conditioning. Heat transfer from the extruded filament to the surrounding atmosphere differs depending on the number and density of filaments in the air gap. Heat transfer is expected to be slower in multifilament spinning. As shown in Figure 4a, temperature simulation along the fiber axis demonstrates that the temperature decreases more rapidly in monofilament spinning. Furthermore, there is variation in humidity within the air gap between monofilament and multifilament spinning, as depicted in Figure 4b. In multifilament spinning, the humidity of the denser filament bundle is significantly lower compared to that in single-filament spinning. The presence of multiple filaments affects the enclosed air, thereby mitigating the impact of external conditions, as evidenced by the simulated temperature and moisture distributions in the air gap area (Figure S2, Supporting Information).

Figure 4c compares the toughness and elongation of fibers produced through monofilament and multifilament spinning using a 400-hole circular spinneret. Interestingly, the fibers obtained from monofilament spinning consistently exhibit higher toughness compared to those obtained from multifilament spinning. One possible explanation for this observation is that the temperature of the fiber during multifilament spinning is significantly higher than during monofilament spinning. This elevated temperature leads to a decrease in viscosity (as shown in Figure 1a) and subsequently reduces the alignment of cellulose molecules along their molecular axes. This phenomenon is consistent with findings from our previous study, where high humidity combined with low temperature in the air gap resulted in the production of high-toughness fibers.^25^

The evaluation of spinnability is based on the minimum achievable titer, with a smaller titer indicating a more stable spinning process. A standard fine textile fiber typically has a titer of 1.3 dtex. As summarized in Table 2, all the spinning trials using [mTBDH][OAc] resulted in fibers with a titer equal to or less than 1.3 dtex, even when using a spinneret with a very high hole density. Notably, monofilament spinning demonstrated the ability to produce finer fibers using the same pulps, while the spinnability decreased with an increase in hole density in the spinneret. Additionally, the presence of a small amount of water in the IL contributed to an improved spinnability, observed in both single- and multifilament spinning processes.

**Table 2:** Minimum Titer and DR Achieved Using Different Pulps and Spinnerets^a^

	spinneret		HV austrocel				LV austrocel			
	hole number	hole density (hole/cm^2^)	IL with 0.1% water		IL with 3% water		IL with 0.1% water		IL with 3% water	
			minimum titer (dtex) and DR							
S1	1		0.88 ± 0.09	15	0.76 ± 0.04	19	0.86 ± 0.03	17	0.79 ± 0.1	17
C400	400	58	1.08 ± 0.2	13	0.81 ± 0.09	16	1.13 ± 0.13	13	0.95 ± 0.11	14
R400	400	175					1.32 ± 0.11	11		
R504	504	221					1.29 ± 0.14	11		

a S1 is single-filament spinning. C400 is the spinneret with 400 holes distributed in a circular (C) geometry. R400 is the spinneret with 400 holes distributed in a rectangular (R) geometry. 13 wt % of cellulose concentration is used in all the trials.

### Fiber Morphology

**Figure 5:**
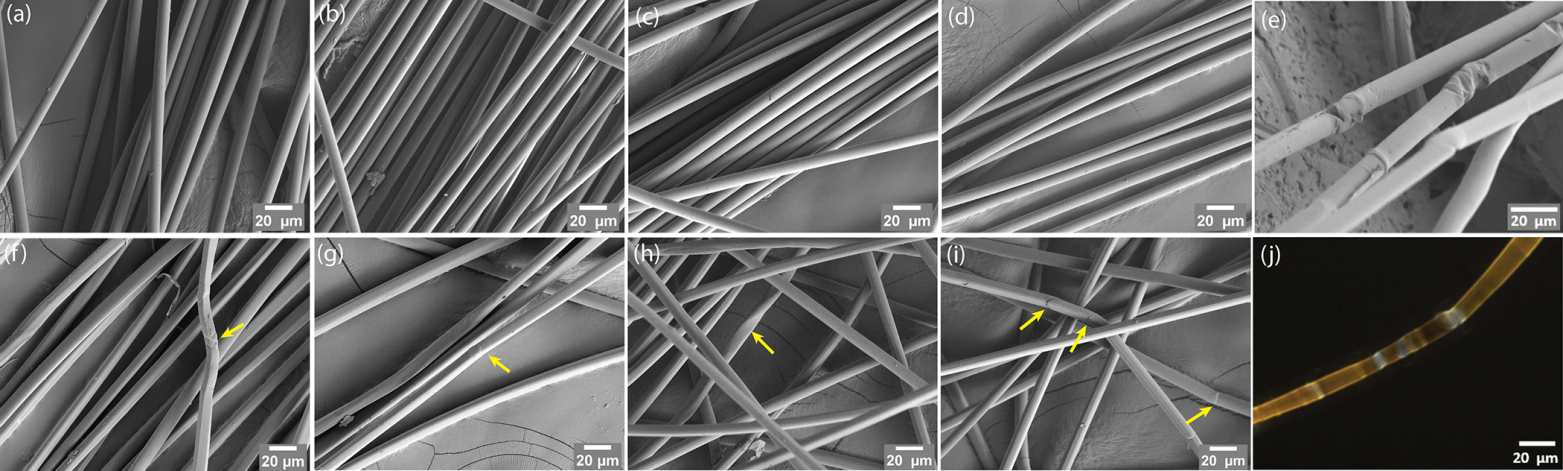
Fiber surface morphology imaged with SEM (a–i) and polarized light microscopy (j). All fibers were collected at DR 11. (a–d) Fibers spun from single filament spinning unit. (e) to (j) fibers spun from multifilament spinning using a circular spinneret with 400 capillaries. (a) and (f) HV austrocel and fresh-prepared IL. (b) and (g) LV austrocel and fresh-prepared IL. (c) and (h) HV austrocel and IL with 3 wt % water. (d) and (i) LV austrocel and IL with 3 wt % water. The yellow arrow points to the kinks of the fibers. (e) Kinks of the fibers (j) cross polarized light microscopy image of the fibers with kinks.

The surface morphologies of fibers spun through single-filament and multifilament spinning methods are analyzed using SEM (Figure 5). The fibers produced via single filament spinning exhibit a very smooth surface, whereas the fibers obtained from multifilament spinning show kink bands, as indicated by yellow arrows in Figure 5e–j. These kink bands can be related to dislocations, which were initially described by Robinson in 1921 during the compression study of wood samples. Under a polarized microscope, the dislocations appear as bright, linear regions of fibers, resulting from the different orientation of cellulose chains or fibrils in the dislocated area.^33^ Subsequently, the presence and characterization of dislocations in natural fibers, such as hemp, have been confirmed by several reports.^34,35^ The polarized microscope image presented in Figure 5j depicts the difference in cellulose chain orientation at the dislocation area compared to the rest of the fiber, as indicated by a distinct color. Tensile testing performed under SEM imaging visualized that the kink bands are prone to initiate cracking. However, the evolution of tensile strength in relation to the number of dislocations per millimeter of the fiber does not exhibit a clear correlation.^36^

When comparing the mechanical properties of fibers spun from single and multifilament spinnerets, we did observe a slight decrease in tensile strength and elongation at break for fibers from multifilament spinning. The presence of kink bands may potentially contribute to the reduction in mechanical properties, but further tests and investigations are necessary to confirm this hypothesis. Dislocations in fibers have significant implications in the chemical treatment and enzymatic hydrolysis of natural cellulose fibers. The kink bands, acting as weak points, provide easier accessibility for chemicals during these processes.^34,37^ It has been reported that the introduction of dislocations to kraft pulps through mechanical treatment can enhance the enzymatic hydrolysis yield of sugars.^37^

## Conclusions

In conclusion, our study demonstrates that the addition of 3 wt % water to the IL significantly enhances the spinnability of fibers while maintaining their mechanical properties. Through monofilament spinning, we achieved high-tenacity fibers at approximately 50 cN/tex with a low titer of 0.7–0.9 dtex. However, when transitioning from single-filament spinning to multifilament spinning, the conditions in the air gap undergo changes that negatively impact spinnability.

The increased number and density of filaments in the air gap restrict heat diffusion and result in elevated temperatures and reduced humidity, posing challenges for the spinning process. It is therefore crucial to carefully adjust the temperature and moisture content in the air gap, particularly when employing high-hole-density spinnerets with a large number of spinning holes, to ensure optimal spinnability.

Despite these challenges, we successfully produced cellulose fibers with a titer of 1.3 dtex, meeting the standard for commercial textile fibers, even when using high-density hole spinnerets without air-gap conditioning (221 holes/cm^2^). In addition, we observed differences in the morphology of fibers spun via single-filament spinning and multifilament spinning. Fibers spun with a 400-hole spinneret displayed more dislocations, which may account for the reduced spinnability observed in multifilament spinning.

## Data Availability

The datasets generated and analyzed during the current study are available from the corresponding author upon reasonable request.

## Supporting Information Available

Geometry of the spinnerets, comsol simulation of the temperature, and moisture distribution at the air gap and angular frequency sweeps of cellulose dopes at 80 °C (PDF)

## Notes

All authors have read and commented on the study. All authors consented to the publication of this work.
