# Supplementary material for: Optimization of Dry-Jet Wet Spinning of Regenerated Cellulose Fibers Using [mTBDH][OAc] as a Solvent
Source: ACS Omega. 2023 Aug 29;8(37):34103–10. doi: 10.1021/acsomega.3c05133 (PMC10515369; doi:10.1021/acsomega.3c05133)
Supplement: Supplementary file 1 — ao3c05133_si_001.pdf [file ao3c05133_si_001.pdf]

Optimization of dry-jet wet spinning of regenerated cellulose fibers using [mTBDH][OAc] as solvent

Wenwen Fang*, E Yee Lim, Kaarlo Nieminen, Herbert Sixta*

*wenwen.fang@aalto.fi, herbert.sixta@aalto.fi

Department of Bioproducts and Biosystems, Aalto University, Vuorimiehentie 1, 02150 Espoo, Finland

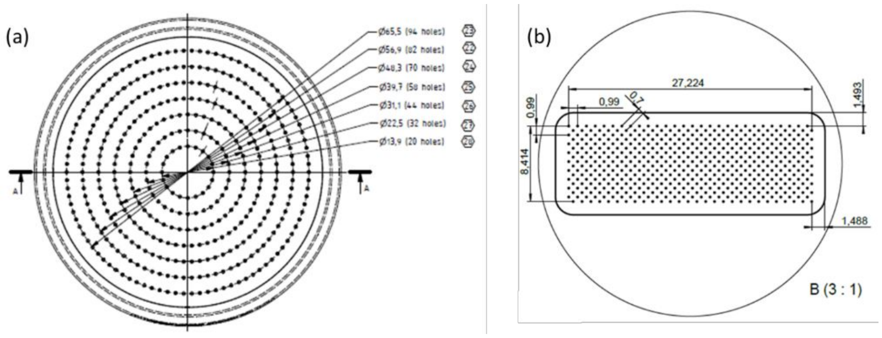

Figure S1. Spinnerets with (a) circular geometry and (b) rectangular geometry.

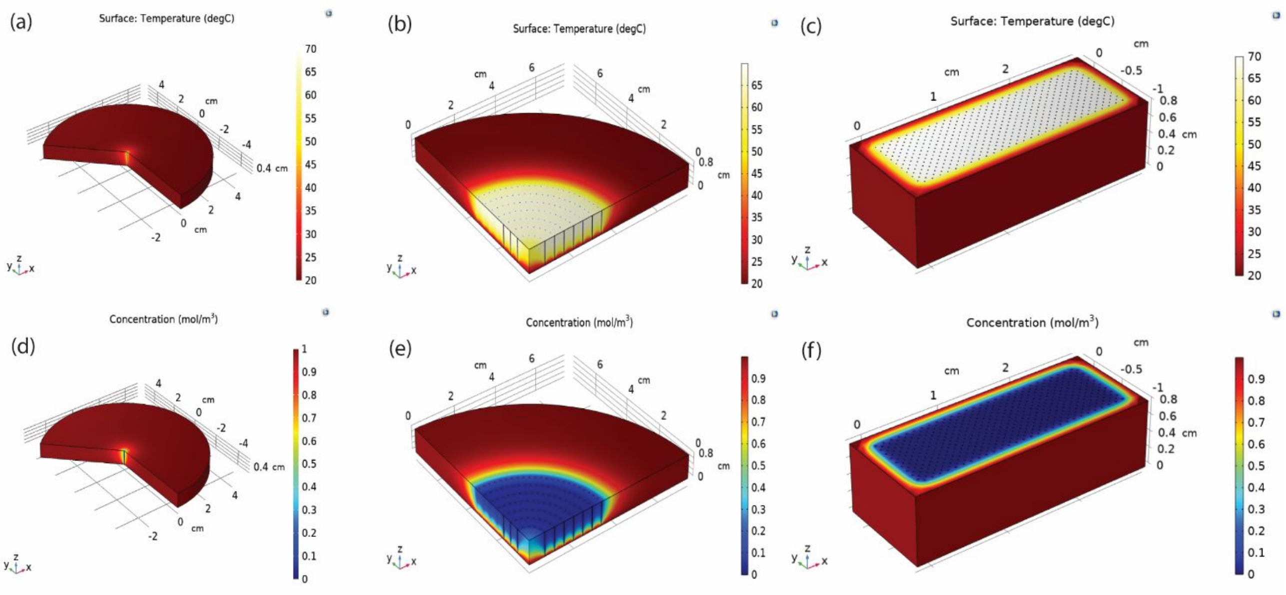

Figure S2. Comsol simulation of the temperature and moisture distribution at the air gap using spinnerets with different hole number and geometry (a) and (d) single filament, (b) and (e) circular spinneret with 400 holes, (c) and (f) rectangular spinneret with 504 holes.

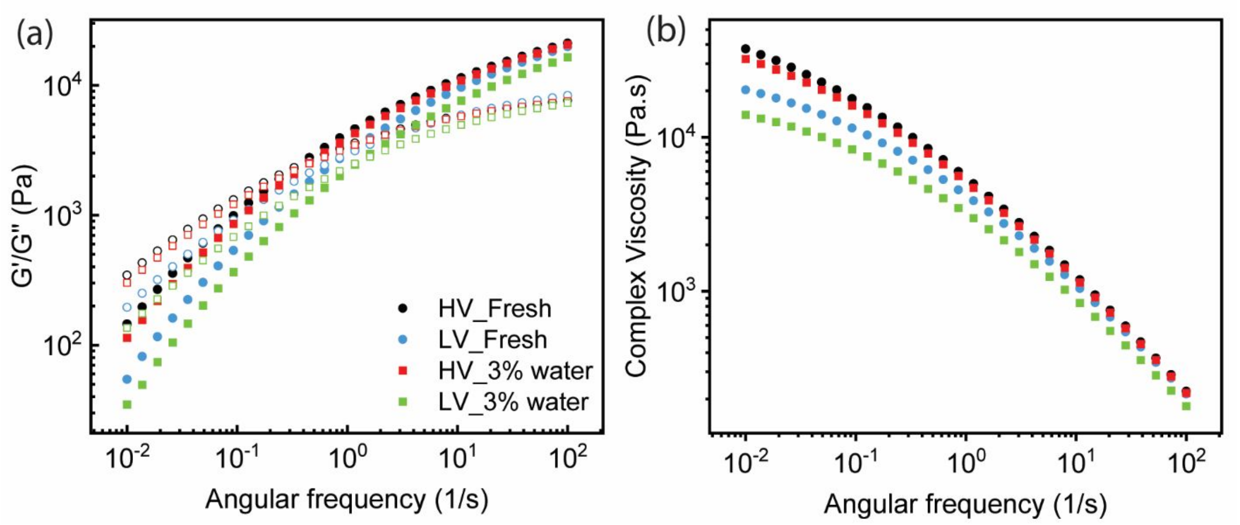

Figure S3. Rheological characterization of (a) Dynamic modulus and (b) complex viscosity of the cellulose dissolutions in [mTBDH] [OAc] as a function of the shear rate at 80°C. Fresh prepared [mTBD] [OAc] with less than 0.1 wt% water and [mTBD] [OAc] with 3 wt% water were used as the solvent. Solid circle symbol is for storage modulus (G') and empty circle symbol is for loss modulus (G").
